# Perceived Respect Climate, Pressure Self-Efficacy, and Intention to Continue Refereeing: Scale Development and a Cross-Sectional Structural Equation Model

**DOI:** 10.3390/healthcare14203476

**Published:** 2026-10-16

**Authors:** Sakir Tufekci, Fatih Harun Turhan, Özgür Eken, Uğur İnce, Mehmet Ozkeskin, Sacide Tufekci, Burak Canpolat, Katja Weiss, Thomas Rosemann, Beat Knechtle

**Affiliations:** 1 Department of Sport Management, Faculty of Sport Sciences, Inonu University, 44280 Malatya, Türkiye; sakir.tufekci@inonu.edu.tr; 2 Department of Sport Management, Hasan Dogan Faculty of Sport Sciences, Karabük University, 78800 Karabük, Türkiye; fharunturhan@karabuk.edu.tr; 3 Department of Physical Education and Sport Teaching, Faculty of Sport Sciences, Inonu University, 44280 Malatya, Türkiye; ozgureken86@gmail.com; 4 Mehmet Arabacı Physical Education and Sport, Harran University, 63000 Şanlıurfa, Türkiye; ugurince@harran.edu.tr; 5 Karabük Youth and Sport Ministry, 78000 Karabük, Türkiye; meteozkeskin@hotmail.com; 6 Department of Physical Education and Sport Teaching, Faculty of Sport Sciences, Kahramanmaras Sutcu Imam University, 46050 Kahramanmaraş, Türkiye; sacidetufekci@ksu.edu.tr; 7 Department of Physical Education and Sport for Disabled, Faculty of Sport Sciences, Inonu University, 44280 Malatya, Türkiye; burak.canpolat@inonu.edu.tr; 8 Institute of Primary Care, University of Zurich, 8091 Zurich, Switzerland; katja@weiss.co.com (K.W.); thomas.rosemann@usz.ch (T.R.); 9 Medbase St. Gallen Am Vadianplatz, Vadianstrasse 26, 9001 St. Gallen, Switzerland

**Keywords:** sport officiating, continuation intention, respect climate, referee self-efficacy, psychometrics, structural equation modelling

## Abstract

**Highlights:**

**What are the main findings?**

- Perceived referee respect climate was strongly associated with intention to continue officiating, whereas the statistical indirect association through pressure self-efficacy was small.
- The newly developed five-item Referee Respect Climate Scale and six-item Referee Continuation Intention Scale showed promising initial psychometric properties.

**What are the implications of the main findings?**

- Respectful and supportive officiating environments are a plausible target for future referee retention research and organisational interventions.
- Independent validation and longitudinal follow-up are required before causal mediation or actual-retention effects can be inferred.

**Abstract:**

Background/Objectives: Retaining qualified referees is essential to organised sport, yet the social and psychological factors associated with referees’ intention to continue remain insufficiently understood. This study developed two referee-specific measures—the Referee Respect Climate Scale (RRC) and Referee Continuation Intention Scale (RCI)—and estimated the association between perceived respect climate and continuation intention, including the statistical indirect association through self-efficacy for performing under pressure. Methods: An online cross-sectional survey was completed by 630 referees between 31 October and 25 December 2025; 625 eligible adults were analysed (67.7% men; mean age: 33.56 ± 10.35 years). The sample was split for exploratory (*n* = 312) and confirmatory (*n* = 313) factor analyses of the newly developed measures. Because Mardia’s test indicated multivariate non-normality, the three-factor measurement and structural models were estimated with robust (Satorra–Bentler) maximum likelihood and 5000-resample bias-corrected bootstrap confidence intervals; sensitivity analyses used ordinal WLSMV estimation, covariate adjustment and multigroup invariance testing by sex. Results: The RRC yielded a five-item unidimensional structure (α = 0.82; CR = 0.82), and the RCI a six-item unidimensional validation model (α = 0.92; CR = 0.92). The measurement model fit well (robust CFI = 0.991, TLI = 0.989, RMSEA = 0.032, SRMR = 0.034). RRC was associated with RCI (β = 0.554) and pressure self-efficacy (β = 0.276), while pressure self-efficacy was associated with RCI (β = 0.117). The indirect association was small (β = 0.032; 95% BC CI: [0.010, 0.064]); the R^2^ for RCI was 0.356. Ordinal, covariate-adjusted and alternative-item sensitivity analyses preserved the substantive pattern. Loading and intercept invariance across sex was supported, whereas ordinal threshold invariance was only approximate. Exploratory multigroup tests suggested a larger pressure self-efficacy path among women, although this was test-dependent. Conclusions: Perceived respect climate was strongly associated with referees’ continuation intention, whereas the indirect association through pressure self-efficacy was small. The new measures show promising initial psychometric properties, but independent and longitudinal validation is required.

## 1. Introduction

Referees occupy a foundational role in ensuring that sport competitions are conducted fairly, safely and in accordance with the rules [1]. Beyond decision making, rule enforcement and match management, referees work in continuous interaction with athletes, coaches, spectators, fellow officials and sport organisations. Officiating is, therefore, not merely a technical task but a socially evaluated work role characterised by time pressure and performance expectations [2]. Maintaining an adequate officiating workforce is important for sport-system sustainability, but intention to continue should be distinguished from observed retention behaviour: the former is a proximal attitudinal outcome, whereas the latter requires longitudinal evidence of continued participation. Understanding factors associated with continuation intention is, nevertheless, important because shortages and attrition remain prominent concerns across countries and sports [3,4].

A substantial portion of the research explaining referees’ intention to remain in the profession has focused on stress, burnout and the inherently demanding conditions of officiating [5]. Although these variables constitute important components of the officiating experience, explaining referees’ retention decisions solely through negative experiences offers a limited perspective [6]. Accordingly, recent research has directed greater attention to the quality of the social and organisational environment in which referees operate, to perceived organisational support, interpersonal relationships, satisfaction and a sense of belonging [7,8,9,10]. Indeed, the support and respect referees encounter in their working environment have been reported to shape their officiating experiences positively and to be associated with their tendency to continue [8,9].

Referees’ professional continuity may, thus, be related not only to individual stress levels but also to the quality of the social environment in which they work [7,11,12]. At this point, referees’ perceptions of the extent to which they are valued and respected in their working environment carry particular importance [13]. A respect-based environment can be characterised by referees’ contributions being valued, by their receiving support during their duties, and by their interactions with other actors occurring within a positive social climate [13,14,15]. The respect referees receive from athletes, coaches and spectators, together with the negative experiences they encounter during matches, has been reported to influence their intention to continue officiating [9].

Organisational Support Theory provides one rationale for expecting social-context perceptions to relate to continuation intentions: individuals develop general beliefs about whether an organisation values their contributions and cares about their well-being, which can shape work-related attitudes and behaviour [16]. Self-Determination Theory offers a complementary account through the need for relatedness and its relevance to motivation and well-being [17]. The respect-climate construct examined here is not treated as synonymous with perceived organisational support, however, because it extends beyond the formal organisation to interactions with coaches, athletes, spectators and other match officials. It is, therefore, conceptualised as a context-specific perception of the interpersonal officiating environment.

Referees’ capacity to carry out their duties effectively under demanding competitive conditions is also related to the psychological resources available to them [18]. Self-efficacy refers to an individual’s belief that they can successfully execute the behaviours required in a given situation [19]. In the officiating context, self-efficacy is treated as an important psychological resource reflecting referees’ belief that they can successfully meet the demands of the role [20,21]. Competence under pressure in particular is important for referees’ ability to sustain performance in demanding match conditions [21,22,23].

Taken together, the literature indicates that referees’ continuation intentions are associated not only with adverse experiences such as stress and burnout but also with perceived organisational support, social relationships, satisfaction and psychological resources [7,8,9,13,24]. However, integrated examinations of the immediate social officiating environment and individual efficacy beliefs remain limited. In particular, a brief referee-specific measure focused on perceived respect, support and interpersonal interaction quality across the officiating environment remains underdeveloped, and it is unclear whether pressure self-efficacy accounts for any appreciable portion of the cross-sectional association between such a climate and continuation intention. Addressing this gap requires both transparent initial psychometric evaluation of the focal measures and cautious structural modelling that does not infer temporal mediation from same-wave data.

Accordingly, this study had two principal aims. First, it sought to develop and obtain initial psychometric evidence for the Referee Respect Climate Scale (RRC) and the Referee Continuation Intention Scale (RCI). Second, it estimated a theory-informed cross-sectional structural model linking perceived respect climate, self-efficacy for performing under pressure and intention to continue refereeing. The directional paths were specified to represent the hypothesised relational structure; they were not interpreted as evidence of temporal or causal ordering. The following hypotheses were formulated:

**H1.**  *Referee respect climate is positively associated with intention to continue refereeing.*

**H2.**  *Referee respect climate is positively associated with self-efficacy for performing under pressure.*

**H3.**  *Self-efficacy for performing under pressure is positively associated with intention to continue refereeing.*

**H4.**  *A positive statistical indirect association between referee respect climate and continuation intention through self-efficacy for performing under pressure will be observed.*

## 2. Materials and Methods

### 2.1. Design and Participants

This study employed a cross-sectional, correlational survey design. Structural equation modelling (SEM) was the analytical (modelling) approach used to estimate the hypothesised measurement and structural relations within this design; it is not itself a research design [25]. Participants were reached through convenience sampling via an online survey link. The link to the Google Forms questionnaire was shared, through the chairs of the provincial referee boards, in WhatsApp groups consisting only of active referees registered with these boards; it was not sent to individuals who were no longer officiating. Eligibility, therefore, required active referee status, operationalised as membership in these board-administered groups, and an age of 18 years or older. Individual licence records were not checked in the anonymous survey; age eligibility was verified from the self-reported age field (see below). In the theory-informed structural specification, referee respect climate (RRC) was treated as the exogenous construct, intention to continue refereeing (RCI) as the endogenous construct, and self-efficacy for performing under pressure (PRS) as the intermediate construct. Direct and indirect paths were estimated to characterise the covariance structure. Because all constructs were measured at the same time point, the model was interpreted associationally; the terms exogenous, endogenous and indirect effect refer to SEM specification and do not establish temporal precedence or causality.

Responses were obtained from 630 referees. Applying the pre-specified inclusion criterion (aged 18 years or older), four participants were excluded because their recorded ages were below 18 years and one because the age field contained a non-numeric entry, yielding an analytic sample of 625 referees. No missing values were present in the focal questionnaire items used in the final psychometric and structural analyses. Participants had a mean age of 33.56 years (SD = 10.35; range: 18–65); 67.7% (*n* = 423) were men and 32.3% (*n* = 202) were women, with respondents drawn from 63 provinces of Türkiye after harmonising spelling/case variants in the free-text province field (624 participants reported province of residence; one response was blank). Among those who reported their sport, volleyball (*n* = 184; 29.4%), basketball (*n* = 97; 15.5%), football (*n* = 87; 13.9%) and handball (*n* = 52; 8.3%) were represented. The sport item, which was not set as a required field in the online form, was left blank by 188 participants (30.1%), and 17 participants (2.7%) reported other, mainly individual, sports (badminton, taekwondo, table tennis, swimming, cycling, shooting, wushu/kickboxing and chess, including multi-sport entries); consequently, sport-specific inference was not attempted. Participant characteristics are presented in Table 1.

### 2.2. Measures

Measurement strategy: The measurement model combined two brief measures developed in this study (RRC and RCI) with one established subscale (PRS). Both new measures were developed within the same planned framework—construct definition and item generation from the literature, qualitative expert review, exploratory factor analysis in a calibration half and confirmatory factor analysis in a validation half—while the established PRS subscale was evaluated confirmatorily. For the RRC, however, the removal of original item 2 was initially informed by a full-sample CFA, so that the specific item selection step was not independent of the validation data. This departure is stated explicitly, and its consequences are evaluated in the Results and Supplementary Materials using repeated random-split calibration/validation analyses and alternative-item sensitivity models. The three measures were then combined in one three-factor measurement model that was evaluated before any structural paths were estimated, in line with the two-step approach [26].

#### 2.2.1. Referee Respect Climate Scale (RRC)

The Referee Respect Climate Scale was developed within the present study to measure perceived respect, support and quality of social interaction in the officiating environment. The relevant literature was reviewed, an item pool was generated, and the draft item pools of both new measures were evaluated by three experts: a professor of sport sciences with previous refereeing experience and two members of referee committees. The experts evaluated each item for its relevance to the construct definition, the clarity of its wording and its suitability for the officiating context [27,28]. In line with their feedback, the wording of two items was simplified, and one item was revised with respect to its content coverage. Expert judgements were recorded qualitatively; content validity indices (CVI/CVR) were not calculated and are, therefore, not claimed as evidence. Both draft item pools were covered by this qualitative expert review. No separate referee pilot study or pretest was conducted for either scale; consequently, pre-field item checking is limited to the qualitative expert review described above.

The initial form comprised six items rated on a five-point Likert scale (1 = strongly disagree, 2 = disagree, 3 = undecided, 4 = agree, and 5 = strongly agree). No items were reverse-scored. Higher scores indicate that referees perceive greater respect and support in their working environment. Exploratory and confirmatory evidence for the scale is reported in the Results; the final form comprises five items. The full item set is provided in Appendix A (Table A1).

#### 2.2.2. Referee Continuation Intention Scale (RCI)

The Referee Continuation Intention Scale was developed within the present study to assess individuals’ intention to continue in the officiating role. An item pool was generated from the relevant literature, and item relevance and clarity were reviewed qualitatively through expert opinion. As with the RRC, no quantitative content validity index was available; the psychometric evidence presented here should, therefore, be regarded as initial rather than definitive validation.

The survey form contained ten candidate items: six continuation-keyed items, for which agreement indicates a stronger intention to continue, and four attrition-keyed items concerning exhaustion, disengagement and plans to quit (Appendix A, Table A2 and Table A3). Keying and wording are not identical: RCI3 (“Even difficult matches do not increase my desire to quit”) contains a negation but is keyed in the continuation direction, and it loaded with the other continuation items (Table S1). Exploratory factor analysis indicated that the ten items did not form a single dimension: the continuation-keyed items clustered on one factor and the attrition-keyed items on another (see Section 3). Because this separation may reflect substantive differences between continuation and attrition and/or wording-related method variance, the present model operationalised continuation intention using only the six continuation-keyed items. The attrition-keyed items were not reverse-scored into the RCI total and were not included in the structural model. This data-informed operational decision requires replication in an independent sample. Items are rated on a five-point Likert scale (1 = strongly disagree; 5 = strongly agree), with higher RCI scores indicating stronger intention to continue refereeing.

Construct coverage: Consistent with conceptualisations of intention as a person’s readiness and planned commitment to perform a behaviour [29] and with prior work on officials’ intention to continue [8], the RCI was designed to capture approach-oriented facets of continuation rather than only a stated plan. RCI1 and RCI5 express the plan to remain active (in the long term and next season), and RCI3 expresses persistence despite adverse match experiences. RCI2 (refereeing as a meaningful part of life) reflects the personal importance that sustains a continuation intention; RCI4 (seeing oneself progressing) reflects a future-oriented career projection within officiating; and RCI6 (wishing to take on more matches when time allows) reflects willingness to maintain or extend involvement. These three items, therefore, broaden the construct towards commitment- and career-related aspects of continuation. All six items loaded strongly on a single factor, but this breadth means that the RCI may overlap with related constructs such as occupational commitment; its discriminant validity against such constructs remains to be tested (Section 4).

#### 2.2.3. Referee Self-Efficacy Scale—Pressure Subscale (PRS)

To measure the pressure construct, the pressure subscale of the Referee Self-Efficacy Scale (REFS) developed by Myers et al. [21] was used in the 13-item, four-factor Turkish form (game knowledge, decision making, pressure and communication) examined by Unutmaz and Gençer [30] in active football referees, which retains the factor structure of the original instrument; an alternative Turkish adaptation [31] extended the scale to 18 items across five factors and was, therefore, not used. This subscale measures referees’ beliefs about their competence to perform under pressure during their duties.

The REFS comprises 13 items across four subscales (game knowledge, decision making, pressure and communication); only the three items of the pressure subscale were used here. These items assess referees’ perceived competence in remaining unaffected by pressure originating from players, spectators and coaches. In the raw survey, the subscale used four ordered response categories (1 = low, 2 = moderate, 3 = high, and 4 = complete) and contained no reverse-scored items. This corresponds to the final REFS scoring structure: Myers et al. [21] administered a five-category scale but, because the lowest category was not endorsed at all for two items, collapsed the first category into the second across all items, yielding the four categories on which the final REFS is scored (low, moderate, high and complete confidence); the Turkish labels used here (az, orta, yüksek, and tam) are direct equivalents. Unutmaz and Gençer [30] report the 13-item four-factor structure in a sample of male football referees; the present sample is 32.3% women and is drawn mainly from volleyball, basketball, football and handball, so that adaptation evidence does not cover the present population, and the within-sample confirmatory evidence reported below carries the weight here (see Section 4). Because the present study used only the three pressure items, confirmatory evidence from the present sample (standardised loadings 0.89–0.95; α = 0.944) is reported in the Results as the primary support for the subscale. Higher scores indicate greater perceived self-efficacy for performing under pressure.

Because a single-factor model with three indicators is just-identified (df = 0), fit indices cannot be computed for this subscale in isolation; its psychometric adequacy was therefore evaluated within the three-factor measurement model presented in the Results. In the present sample, internal consistency was α = 0.944.

### 2.3. Procedure and Ethical Approval

All stages of this research were conducted in accordance with the Declaration of Helsinki. Ethical approval was granted by the Social and Human Sciences Research Ethics Committee of Karabük University (meeting date: 31 October 2025; meeting no. 2025/10; decision no. 58; document no. E-78977401-050.04-473974). Because no separate pilot sample was recruited and no pilot data were collected, there was no separate pilot-specific ethics approval or consent procedure; the ethics approval reported here pertains to the main cross-sectional survey.

Data were collected through an online questionnaire from volunteer referees reached by convenience sampling. Responses were recorded between 31 October and 25 December 2025. The questionnaire took approximately 10 min to complete. The first page explained the purpose of this research, and written informed consent was obtained from all participants. Personal information was kept confidential, and anonymity was ensured. Recruitment primarily targeted referees in team sports such as football, handball, volleyball and basketball; however, the responding sample also contained participants with unreported or other/individual-sport classifications, which were retained in the overall analysis and described transparently.

Individuals under 18 years of age were not included. Two reasons underpinned this criterion: first, the adaptation studies of the instruments used were conducted with individuals aged 18 and over; second, restricting the sample to adults removed the requirement for parental consent.

### 2.4. Data Analysis

Descriptive statistics were computed in SPSS 27. All factor-analytic, SEM, bootstrap, invariance and sensitivity analyses described in this study were conducted in R 4.3.3 [32] using lavaan 0.6-17 [33], semTools 0.5-6 [34] and psych 2.4.1 [35], with oblique rotations obtained through its GPArotation dependency; the maximum-likelihood solution obtained in AMOS 24 was reproduced exactly in lavaan (χ^2^(74) = 178.61). The complete R script and model syntax are provided as Data S1 (Data_S1_analysis_script.R), together with the case-level calibration/validation membership file (Data_S1_split_assignment.csv); the documented random seed is 20260921, and the script reads the split membership from that file so that the calibration and validation analyses reproduce independently of the random-number implementation. First, descriptive statistics and diagnostics for univariate distributional shape, linearity and multicollinearity (VIF; tolerance) were examined [36]. Multivariate normality was assessed with Mardia’s multivariate skewness and kurtosis coefficients [37], and multicollinearity was assessed both at the construct level (inner VIF) and at the item level (outer VIF; each indicator regressed on the other indicators of its construct) [38]. The HTMT ratio and item-level (outer) VIFs originate in variance-based (PLS) structural equation modelling and are included as descriptive diagnostics alongside the covariance-based criteria. Second, for the two measures developed in this study, the sample was randomly split into two halves (seed 20260921): exploratory factor analysis (EFA) using principal axis factoring with parallel analysis (1000 random datasets [39]) and oblimin rotation when more than one factor was retained was conducted in the calibration half (*n* = 312), and confirmatory factor analysis (CFA) was performed in the validation half (*n* = 313). Because the decision to remove one RRC item had originally been informed by a full-sample CFA, its stability was evaluated in 1000 repeated random splits of the same 625-participant dataset. Within each calibration half, the item removal rule selected the single deletion that yielded the lowest normal-theory ML χ^2^ (all candidate five-item models have df = 5, so this is the largest χ^2^ reduction); the resulting five-item form was then evaluated in the held-out half. This is an internal resampling-based stability analysis, not validation in a new sample, and it does not undo the original use of the full sample for item selection. Third, the three-factor measurement model was estimated in the total analytic sample (*n* = 625); convergent evidence was evaluated using standardised factor loadings, composite reliability (CR) and average variance extracted (AVE), and discriminant evidence was evaluated using the Fornell–Larcker criterion [40] and HTMT ratios [41] with 95% percentile bootstrap confidence intervals (5000 resamples). Fourth, the structural model was estimated, with direct, indirect and total associations accompanied by 5000-resample bias-corrected bootstrap confidence intervals. Bias-corrected intervals are reported for the structural estimates and percentile intervals for the HTMT ratios, following the conventions usually applied to each; because bias-corrected intervals can be liberal in tests of indirect associations, the small indirect estimate reported below should be read with that in mind. Because Mardia’s test indicated multivariate non-normality (Section 3.1), the measurement and structural models were estimated by maximum likelihood with Satorra–Bentler scaled test statistics and robust standard errors (MLM) [42], and robust CFI, TLI and RMSEA values are reported [43,44]; point estimates are identical to those of normal-theory ML. Unstandardised estimates (B) with robust standard errors and z- and *p*-values are reported separately from standardised coefficients (β) with bootstrap standard errors and bias-corrected confidence intervals. Fifth, two nested constrained specifications were compared using chi-square difference tests (Satorra–Bentler scaled difference tests [45]). Sixth, post hoc robustness analyses were performed: (i) an ordinal re-estimation treating all indicators as ordered categorical (polychoric correlations; WLSMV estimation with mean- and variance-adjusted test statistics [46]), with delta-method confidence intervals for standardised paths and a Monte Carlo interval (20,000 draws) for the indirect association [47]; (ii) multigroup CFA by sex testing configural, metric and scalar invariance under MLM with scaled χ^2^-difference tests and changes in robust CFI [48], supplemented by an ordinal (WLSMV) sequence testing the equality of thresholds, loadings and intercepts with the identification conditions of Wu and Estabrook [49]; (iii) group-specific structural estimates, all obtained from the scalar-invariant multigroup model as the common reference model, followed by single equality constraints on each structural path and on the unstandardised indirect product a × b; (iv) covariate-adjusted models including age, sex and income (*n* = 625) and, additionally, officiating experience (*n* = 579 classifiable responses); and (v) models using the full six-item RRC pool or an alternative five-item RRC form. Model fit was evaluated primarily using CFI, TLI, RMSEA and SRMR [50]. Harman’s single-factor test was used only as a descriptive diagnostic and was not treated as a definitive test that could exclude common-method bias [51]. For the experience-adjusted sensitivity model, classifiable officiating experience was coded ordinally as 1, 2, 3 and 4 for 1 year, 2 years, 3 years and 4 or more years, respectively; the 45 placeholder-labelled responses and one blank response were excluded only from that model.

### 2.5. Sample Size and Estimator Considerations

All 625 eligible cases were retained for the main analysis; no formal a priori SEM power calculation was available. Accordingly, interpretation emphasises standardised estimates and confidence intervals rather than statistical significance alone. The split samples (*n* = 312 and *n* = 313) and the full analytic sample were adequate for the limited-indicator models estimated here, but the absence of a design-stage power calculation remains a limitation. Because Mardia’s coefficients indicated marked multivariate non-normality, robust ML (MLM) was adopted as the primary estimator while retaining direct comparability with the normal-theory ML solution. This choice has a known limitation. Rhemtulla et al. [46] recommend categorical estimation for indicators with fewer than five categories and regard robust ML as adequate mainly when items have five or more categories and their thresholds are approximately symmetric; they further show that parameter estimates become increasingly negatively biased as thresholds become more asymmetric. The eleven RRC and RCI items have five categories, but the three PRS items have only four, and the item distributions are markedly skewed (Section 3.1). Both conditions, therefore, favour categorical estimation for the paths involving PRS. Accordingly, the ordinal WLSMV solution is reported in full alongside the primary analysis rather than as a peripheral check (Section 3.7) and the estimator dependence of the indirect association is reported explicitly. The present analyses do not identify the mechanism responsible for the difference between estimators; the smaller PRS-related estimates under robust ML are, therefore, described only as consistent with attenuation patterns discussed by [46], not as confirmation that attenuation caused the observed difference. The ordinal analysis was specified post hoc and is not presented as a prospectively planned primary analysis.

## 3. Results

### 3.1. Preliminary Analyses and Common Method Variance

No missing values were present on any analysed item. At the item level, skewness ranged from −1.99 to −0.79 and kurtosis from +0.27 to +4.78, indicating some departure from normality for several items. Mardia’s coefficients indicated substantial multivariate non-normality (multivariate skewness *b*_1_,*_p_* = 55.71, χ^2^(560) = 5803.21, *p* < 0.001; multivariate kurtosis *b*_2_,*_p_* = 415.81 against an expected value of 224 under normality, z = 113.28, *p* < 0.001); robust ML was, therefore, used for all covariance structure models. Harman’s single-factor test showed that the first unrotated factor accounted for 43.06% of the total variance, and a one-factor CFA fit the data poorly: χ^2^(77) = 2421.90, χ^2^/df = 31.45, CFI = 0.589, TLI = 0.515, RMSEA = 0.221 (robust CFI = 0.592; robust RMSEA = 0.218). These diagnostics provide no evidence of a dominant single factor; however, they do not rule out common-method bias arising from same-source, same-time self-report measurement.

### 3.2. Psychometric Properties of the Developed Scales

Item-level descriptive statistics and exploratory and confirmatory factor loadings for the RRC and RCI scales are presented in Table 2. The initial six-item unidimensional CFA of the RRC in the full analytic sample (*n* = 625) fit the data poorly: χ^2^(9) = 88.46, χ^2^/df = 9.83, CFI = 0.947, TLI = 0.911, RMSEA = 0.119 [0.097, 0.142], SRMR = 0.041. Inspection of residual covariances indicated that item 2 (“Even when players protest, they do not overstep the line”) overlapped in content with other respect-climate items and contributed to local dependence. Among the six possible single-item deletions, removing item 2 also produced the lowest ML χ^2^ (χ^2^(5) = 16.31 vs. 41.37–71.12 for the other deletions) and the highest robust CFI (0.991). Rather than adding correlated residuals, the item was removed, and the five-item form was re-estimated. Because this removal was informed by the observed data as well as content overlap, the resulting structure should be considered provisional and in need of independent replication. The five-item form showed good fit in the subsequent analyses reported below. Because this diagnostic was obtained in the full sample, the validation-half CFA was not fully independent of the item selection decision. To quantify within-sample selection stability, the same decision rule (remove the single item whose deletion yielded the lowest ML χ^2^ in the calibration half) was applied across 1000 repeated random splits: original item 2 was selected in 90.9% of calibration halves (item 6 in 5.3%; each remaining item in ≤1.6%), and the resulting five-item form showed a robust CFI ≥ 0.95 and SRMR ≤ 0.08 in 94.3% of the corresponding held-out halves (median robust CFI = 0.990, TLI = 0.981, RMSEA = 0.055, SRMR = 0.026; Table S2). Because every split was drawn from the same 625-participant dataset, these results describe internal stability and do not constitute external validation or restore independence to the initial full-sample item selection step. In the single seed-documented split used for Table 2 and Table S1, the same rule selected original item 6 rather than item 2 (ML χ^2^(5) = 6.42), reflecting a residual dependence between original items 3 and 6 (residual correlation = 0.13) and illustrating split-to-split variability; nevertheless, the six-item form fit poorly in the corresponding validation half (robust CFI = 0.923; RMSEA = 0.142), whereas the five-item form fit acceptably (below). Retaining all six items, or removing item 6 instead of item 2, left the structural conclusions unchanged, as detailed in Section 3.8.

In the calibration half, sampling adequacy and factorability were satisfactory (KMO = 0.838; Bartlett’s χ^2^(10) = 510.4 (*p* < 0.001)), parallel analysis retained one factor, and that factor accounted for 48.77% of the total variance. In the validation half, the five-item model fitted acceptably, scaled χ^2^(5) = 10.92, robust CFI = 0.983, robust TLI = 0.966, robust RMSEA = 0.073 [0.000, 0.132], SRMR = 0.028 (ML χ^2^(5) = 15.25; Table S3).

For the RCI, parallel analysis on the ten candidate items retained two factors (observed eigenvalues of 5.10 and 0.90 vs. 0.47 and 0.22 for random data). In the oblimin-rotated principal-axis solution (Table S1), the six continuation-keyed items loaded 0.76–0.86 on the first factor and the four attrition-keyed items 0.49–0.89 on the second; the maximum absolute cross-loading was 0.215 (ATT4 = −0.215), and the two factors correlated at r = −0.57. Two attrition items describe exhaustion or strain (“I feel mentally exhausted after matches”; “Lately, refereeing has been wearing me down”), whereas the negated but continuation-keyed RCI3 loaded 0.77 on the continuation factor, suggesting that the separation reflects content (strain and disengagement vs. continuation) at least as much as wording. Because the split may reflect both construct content and item wording, the present study did not interpret the two-factor solution as definitive evidence of two distinct latent traits. Instead, the six continuation-keyed items were used as the operational RCI factor for the planned structural model. In the calibration half, KMO = 0.908 and Bartlett’s χ^2^(15) = 1250.8 (*p* < 0.001), with a single factor accounting for 65.44% of the total variance; in the validation half, the six-item model fit well: scaled χ^2^(9) = 14.09, robust CFI = 0.990, robust TLI = 0.983, robust RMSEA = 0.071 [0.000, 0.139], SRMR = 0.024 (ML χ^2^(9) = 39.25). Across the 1000 random splits, the six-item RCI met robust CFI ≥ 0.95 and SRMR ≤ 0.08 in 99.6% of validation halves (median robust CFI = 0.989; RMSEA = 0.073). Item statistics, factor loadings and item-level VIFs appear in Table 2.

### 3.3. Measurement Model, Reliability and Validity

**Figure 1:**
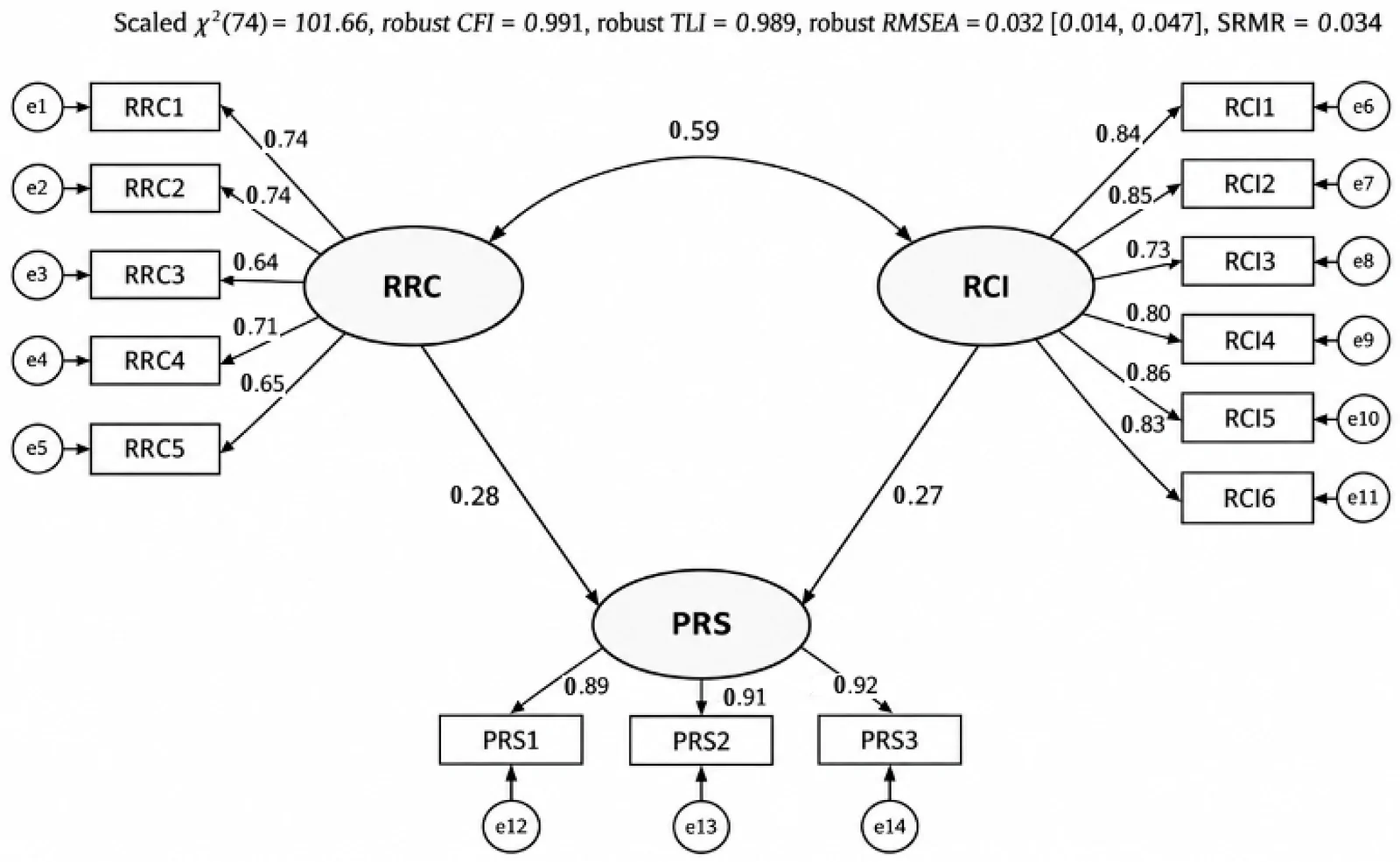
Three-factor measurement model with standardised factor loadings (*N* = 625). Note: RRC = referee respect climate; RCI = referee continuation intention; PRS = self-efficacy for performing under pressure. Double-headed arrows denote latent correlations. All parameters are significant at *p* < 0.001. The fit indices shown are Satorra–Bentler robust values.

The three-factor measurement model (14 items) fit the data well under robust ML: Satorra–Bentler scaled χ^2^(74) = 101.66, *p* = 0.018 (scaling factor = 1.757), robust CFI = 0.991, robust TLI = 0.989, robust RMSEA = 0.032 (90% CI: [0.014, 0.047]), SRMR = 0.034. The corresponding normal-theory ML values were χ^2^(74) = 178.61, χ^2^/df = 2.41, CFI = 0.982, TLI = 0.977, NFI = 0.969, GFI = 0.961, AGFI = 0.944 and RMSEA = 0.048 [0.039, 0.057]. All standardised loadings ranged from 0.64 to 0.95 and were significant at *p* < 0.001 (Figure 1).

Cronbach’s alpha ranged from 0.82 to 0.94 and composite reliability from 0.82 to 0.95 (Table 3). The AVE exceeded 0.50 for RCI and PRS. For RRC, the AVE was 0.484, marginally below the conventional 0.50 benchmark, whereas the CR was 0.824. Fornell and Larcker [40] regard convergent validity as still acceptable when the AVE falls below 0.50, provided that composite reliability exceeds 0.60, which is the case here. Accordingly, convergent evidence for the RRC was considered borderline but broadly supportive rather than unequivocal. Discriminant evidence was favourable under the Fornell–Larcker criterion and HTMT ratios (RRC–RCI = 0.601, 95% CI [0.509, 0.683]; RRC–PRS = 0.279 [0.194, 0.363]; RCI–PRS = 0.281 [0.194, 0.365]); all upper confidence limits were below 0.85. These findings support continued evaluation of the RRC while underscoring the need for independent replication and possible refinement of the item pool.

### 3.4. Descriptive Statistics, Normality, Correlations and Multicollinearity

Respect climate (M = 4.02; SD = 0.72) and continuation intention (M = 4.37; SD = 0.75) were rated highly, whereas pressure self-efficacy was rated at a moderate-to-high level on its four-point metric (M = 3.26; SD = 0.91). Skewness ranged from −1.74 to −1.12 and kurtosis from 0.72 to 4.13 at the scale-score level. Continuation intention showed the greatest kurtosis (+4.13). Together with the significant Mardia coefficients (Section 3.1), these distributions motivated robust ML estimation with bootstrap confidence intervals.

Respect climate correlated moderately with continuation intention (r = 0.52; *p* < 0.001) and weakly with pressure self-efficacy (r = 0.24; *p* < 0.001); pressure self-efficacy and continuation intention were also weakly related (r = 0.26; *p* < 0.001). Because higher scores on the pressure subscale denote greater capability to perform under pressure rather than greater perceived pressure, these positive associations are in the conceptually expected direction. For the predictors, VIF = 1.06 and tolerance = 0.94, indicating no multicollinearity (Table 4). At the item level, the outer VIFs ranged from 1.49 to 1.79 for RRC, 2.07 to 3.35 for RCI, and 3.85 to 5.63 for PRS (Table 2); two RCI items (RCI5 = 3.35 and RCI2 = 3.04) exceed the stricter ideal value of 3 while remaining well below the conventional threshold of 5 applied here, and the value slightly above 5 for PRS2 reflects the near-parallel content of the three pressure items within one construct and does not indicate multicollinearity among the predictors of the structural model.

### 3.5. Structural Model and Indirect Association Analysis

A structural model without the PRS intermediate path was first estimated to characterise the total model-based association between RRC and RCI. It fit well: scaled χ^2^(75) = 107.23, robust CFI = 0.990, robust TLI = 0.988, robust RMSEA = 0.035, SRMR = 0.047 (ML χ^2^(75) = 187.86), with a standardised total association of β = 0.593 (*p* < 0.001; R^2^ = 0.351).

**Figure 2:**
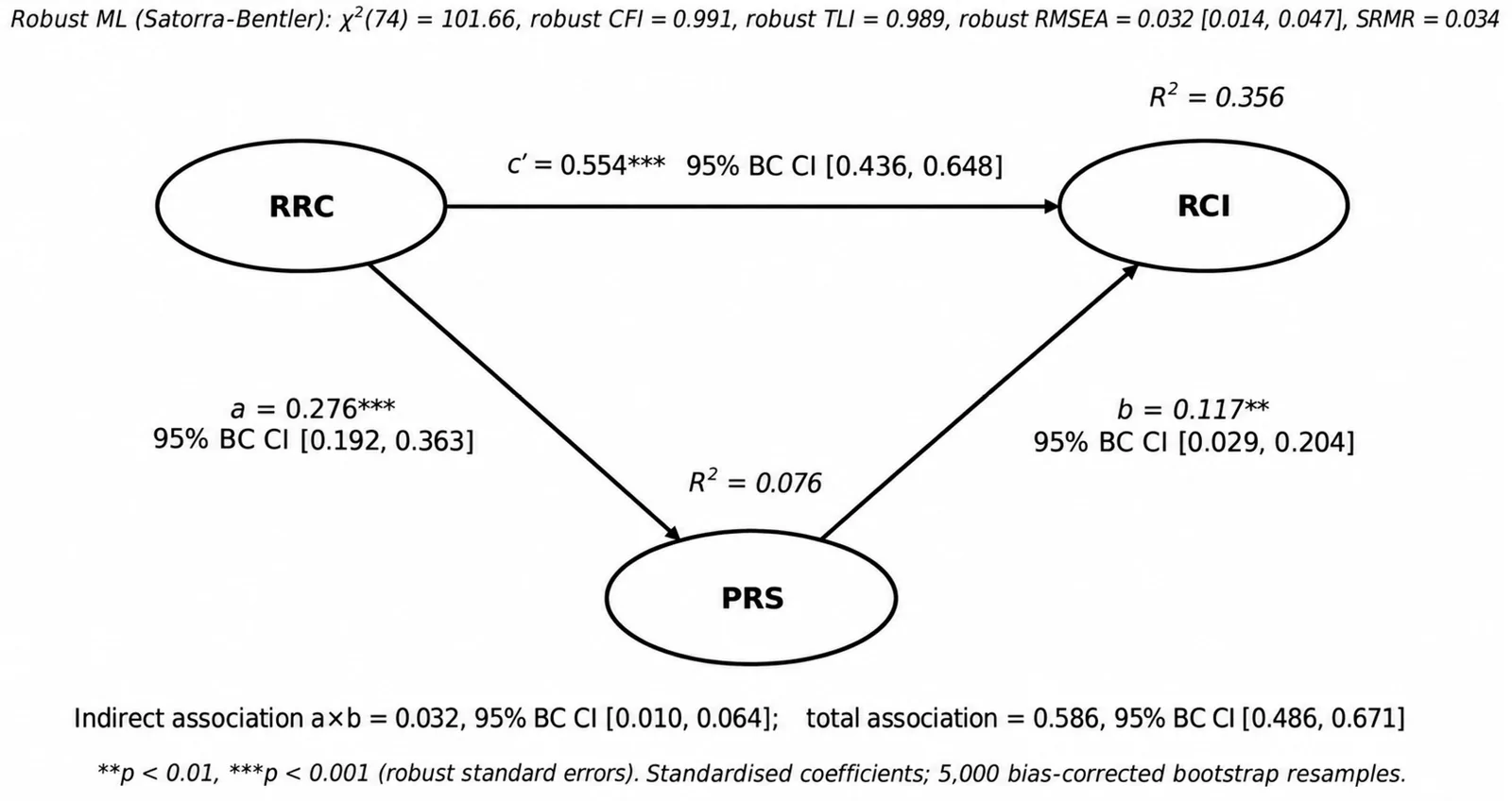
Cross-sectional structural model of respect climate, pressure self-efficacy and continuation intention (*N* = 625). Note. Coefficients are standardised, and confidence intervals are derived from 5000 bias-corrected bootstrap resamples; *p*-values are based on robust standard errors. Arrows represent the specified SEM paths and should not be interpreted as establishing temporal or causal direction. ** *p* < 0.01; *** *p* < 0.001.

The hypothesised cross-sectional indirect association model fit identically to the measurement model (scaled χ^2^(74) = 101.66, robust CFI = 0.991, robust TLI = 0.989, robust RMSEA = 0.032 [0.014, 0.047], SRMR = 0.034). All specified structural paths were statistically different from zero (Figure 2; Table 5). This identity is expected; with three latent variables, the structural part (one path into PRS and two into RCI) is just-identified and simply replaces the three latent covariances of the measurement model with three regression paths. The structural model is, therefore, statistically equivalent to the measurement model; its global fit reflects the measurement model only; and it provides no separate test of the proposed ordering. Alternative orderings of the three constructs would reproduce exactly the same fit [52]. Support for the hypotheses, therefore, rests on the path estimates and their confidence intervals, not on global fit.

Respect climate showed a strong positive direct association with continuation intention (β = 0.554, *p* < 0.001; 95% BC CI: [0.436, 0.648]), supporting H1. Respect climate was also positively associated with pressure self-efficacy (β = 0.276, *p* < 0.001; 95% BC CI: [0.192, 0.363]), supporting H2, and pressure self-efficacy was positively associated with continuation intention (β = 0.117, *p* = 0.008; 95% BC CI: [0.029, 0.204]), supporting H3.

The bootstrapped statistical indirect association was positive, and its 95% bias-corrected confidence interval excluded zero (β = 0.032; 95% BC CI: [0.010, 0.064]), supporting H4. The indirect component represented only 5.5% of the total model-based association, while the direct RRC–RCI path remained substantial. Thus, the data are consistent with a small complementary indirect pathway within the specified cross-sectional model, but they do not establish temporal mediation. The model explained 35.6% of the variance in continuation intention and 7.6% in pressure self-efficacy.

### 3.6. Competing Nested Specifications and Exploratory Sex-Stratified Analyses

Two nested constrained specifications were compared with the hypothesised cross-sectional model to assess whether the direct RRC–RCI path and the PRS–RCI path contributed to model fit. A model constraining the direct RRC–RCI path to zero fit substantially worse: scaled χ^2^(75) = 192.70, robust CFI = 0.963, robust TLI = 0.955, robust RMSEA = 0.067, SRMR = 0.161; scaled Δχ^2^(1) = 64.58, *p* < 0.001. A model constraining the PRS–RCI path to zero also fit worse: scaled χ^2^(75) = 107.23, scaled Δχ^2^(1) = 6.66, *p* = 0.010. These comparisons support retention of both paths within the specified covariance model (Table 6), but they do not establish causal direction or temporal ordering. Because each constrained model differs from M1 by a single structural path, these comparisons are equivalent to tests of the corresponding coefficients and, like global fit, cannot discriminate between alternative orderings of the constructs.

As a descriptive precursor to formal multigroup testing, the model was estimated separately for men (n = 423) and women (n = 202). The measurement model fit acceptably in both groups (men: ML χ^2^(74) = 158.26, scaled χ^2^(74) = 86.65, robust CFI = 0.994, robust TLI = 0.993, robust RMSEA = 0.027, SRMR = 0.038; women: ML χ^2^(74) = 131.14, scaled χ^2^(74) = 91.11, robust CFI = 0.986, robust TLI = 0.982, robust RMSEA = 0.041, SRMR = 0.047). Estimated with the same likelihood normalisation, the two group-specific ML χ^2^ values sum exactly to the configural multigroup ML χ^2^ (158.255 + 131.137 = 289.392, printed as 289.39 in Table 7; adding the two-decimal values shown above gives 289.40 through rounding alone). The configural multigroup value is, therefore, numerically consistent with the group-specific estimates. Group-specific structural estimates with p values and 95% confidence intervals, all obtained from the scalar-invariant multigroup model as the common reference model, are reported in Section 3.7.

The sex-specific structural estimates obtained from the scalar-invariant model are presented in Table 8.

### 3.7. Ordinal Sensitivity and Multigroup Invariance Analyses

In the post hoc ordinal re-estimation (polychoric correlations, WLSMV), the model fit well (scaled χ^2^(74) = 181.32, CFI = 0.995, TLI = 0.994, RMSEA = 0.048 [0.039, 0.057], SRMR = 0.034), and standardised loadings ranged from 0.674 to 0.971. The structural pattern was preserved: RRC → PRS β = 0.404, 95% CI: [0.324, 0.483]; PRS → RCI β = 0.176 [0.089, 0.262]; direct RRC → RCI β = 0.554 [0.478, 0.630]; indirect association β = 0.071 [0.034, 0.108] (Monte Carlo CI [0.035, 0.109]); total association β = 0.625 [0.566, 0.683]; R^2^ = 0.163 for PRS and 0.416 for RCI (see Section 3.8). WLSMV served as the ordinal sensitivity estimator. Relative to the primary ML solution, treating the indicators as ordinal increased the smaller a and b paths but left the direct RRC → RCI path virtually unchanged. Thus, the substantive conclusion—a dominant direct association accompanied by a smaller indirect component—was robust to this estimator sensitivity check. The size of the indirect estimate, however, was clearly estimator-dependent; it was more than twice as large under WLSMV (0.071) as under ML (0.032), with only partly overlapping confidence intervals. The larger ordinal estimate is compatible with attenuation patterns described for coarse or asymmetric ordinal indicators [46], but the present sensitivity analysis does not establish that attenuation is the cause of the estimator difference. The two standardised indirect-association point estimates were 0.032 under robust ML and 0.071 under WLSMV (point-estimate range: 0.032–0.071); their confidence intervals are reported separately above. Under either estimator, the indirect component remained small in absolute terms and accounted for 5.5% (ML) and 11.4% (WLSMV) of the total association, respectively. The standardised structural estimates from the primary and sensitivity analyses are summarised in Table 9.

Under robust ML, which treats the responses as continuous, loading (metric) and intercept (scalar) invariance across sex were supported (metric: scaled Δχ^2^(11) = 5.58, *p* = 0.900, Δrobust CFI = +0.002; scalar: scaled Δχ^2^(11) = 15.40, *p* = 0.165, Δrobust CFI = −0.001; Table 7A). Because these tests do not evaluate whether the ordinal response thresholds are equal, an ordinal invariance sequence was added (Table 7B). Constraining the thresholds to equality produced a statistically significant scaled Δχ^2^(14) = 29.58, *p* = 0.009, although the change in CFI was negligible (−0.001), and univariate score tests identified no single threshold constraint as a significant source of misfit (all *p* ≥ 0.06); subsequent equality of loadings (Δχ^2^(11) = 5.88; *p* = 0.882) and intercepts (Δχ^2^(11) = 12.43; *p* = 0.332) did not worsen fit. Measurement comparability across sex is, therefore, supported for loadings and intercepts, whereas threshold invariance is only approximate, and comparisons across sex should be interpreted with this qualification.

Group-specific structural estimates were obtained from the scalar-invariant robust-ML model, which served as the reference model for all between-group comparisons (Table 8). Respect climate was positively associated with continuation intention and with pressure self-efficacy in both groups, and equality constraints on these two paths did not worsen fit. The PRS → RCI path was small and not statistically significant among men (B = 0.060, 95% CI [−0.042, 0.163], *p* = 0.245; β = 0.063) but was significant among women (B = 0.219 [0.095, 0.342], *p* = 0.001; β = 0.247), and constraining this unstandardised path to equality worsened fit (scaled Δχ^2^(1) = 6.86; *p* = 0.009). Correspondingly, the indirect association was not significant among men (β = 0.018 [−0.013, 0.048]) but was significant among women (β = 0.068 [0.005, 0.131]), and constraining the unstandardised indirect products to equality worsened fit (scaled Δχ^2^(1) = 8.55; *p* = 0.003). However, the Wald-type confidence intervals for the between-group differences marginally included zero (ΔB for PRS → RCI = 0.158 [−0.001, 0.317]; Δ(a × b) = 0.068 [−0.025, 0.161]), so the evidence for a sex difference depended on the test used. The three tests of the same constraint are not equivalent in finite samples: for the PRS → RCI path, the normal-theory likelihood-ratio difference was Δχ^2^(1) = 4.52, the Satorra–Bentler scaled difference test value was 6.86, and the corresponding Wald statistic, computed from the robust standard errors of the reference model, was 3.80. The scaled difference test rescales the discrepancy between two models whose scaling factors differ, whereas the Wald statistic uses only the sandwich standard errors of the reference model; with markedly unbalanced groups (n = 423 versus 202) and a small coefficient, these statistics can diverge, which is why both are reported rather than either alone. These sex differences were post hoc and should be treated as hypothesis-generating pending independent replication in balanced samples.

### 3.8. Covariate-Adjusted and Alternative-Specification Sensitivity Analyses

Age, sex and income were available for all 625 participants, and officiating experience could be classified for 579 (Table 1). Experience was entered as an ordinal 1–4 covariate corresponding to 1 year, 2 years, 3 years and 4 or more years, respectively. Other potentially relevant variables—competitive level or licence category, match load, remuneration and exposure to abuse—were not collected, and sport was missing for 30.1% of participants. Adjusting all structural paths (and respect climate) for age, sex and income left the estimates virtually unchanged (RRC → RCI β = 0.556, RRC → PRS β = 0.269, PRS → RCI β = 0.117; indirect β = 0.032, 95% CI: [0.006, 0.057]). Additionally, adjusting for officiating experience (n = 579) yielded the same pattern (β = 0.570, 0.246 and 0.105; indirect β = 0.026 [0.002, 0.050]); the corresponding unadjusted estimates in the same subsample were 0.562, 0.267, 0.102 and 0.027 (Table 9). Among the covariates in the fully adjusted model, older age was associated with lower pressure self-efficacy (β = −0.17; *p* = 0.006) and lower perceived respect climate (β = −0.20; *p* < 0.001), more officiating experience was associated with higher perceived respect climate (β = 0.16; *p* = 0.004), and women reported lower pressure self-efficacy than men (β = −0.09; *p* = 0.033); no covariate was significantly associated with continuation intention (Table S4). Finally, using all six RRC items, or removing original item 6 instead of item 2, preserved the substantive pattern, with the direct RRC → RCI association remaining dominant (β = 0.525 and 0.498) and the indirect association remaining small (β = 0.035 and 0.036; Table 9).

Overall, the primary robust-ML model, the ordinal WLSMV re-estimation, the covariate-adjusted and alternative-item models and the multigroup analyses converged on a strong RRC–RCI association and a smaller indirect association through pressure self-efficacy. The exploratory sex comparison suggests that the PRS → RCI component may be more prominent among women, although this depended on the test used, and threshold invariance was only approximate. However, the cross-sectional design still cannot establish temporal mediation, and the sex-specific finding requires replication.

## 4. Discussion

This study contributed two related forms of evidence: initial psychometric evidence for referee-specific measures of perceived respect climate and continuation intention, and a cross-sectional structural model linking respect climate, pressure self-efficacy and continuation intention. The dominant empirical pattern was a strong positive RRC–RCI association, whereas the statistical indirect association through pressure self-efficacy was small. These findings are consistent with the literature emphasising organisational support, social climate and community as correlates of officials’ continuation intentions [8,53,54,55]. Importantly, the present design measures intention rather than observed retention and cannot establish that respect climate precedes either efficacy or subsequent continuation behaviour.

The clearest finding was the association between respect climate and continuation intention. The zero-order correlation was moderate (r = 0.52; *p* < 0.001), and the total standardised RRC–RCI association in the structural model was β = 0.59 (*p* < 0.001). This pattern accords with evidence linking supportive working environments, perceived organisational support, mentoring and fair governance to stronger commitment and lower turnover intention among officials [12,24,56,57,58,59]. The present RRC construct extends this perspective beyond formal organisational support by incorporating perceived respect and support across the immediate officiating environment. Whether interventions that modify this climate subsequently improve actual retention remains an empirical question for longitudinal or experimental work.

After pressure self-efficacy was included, the direct RRC–RCI path remained strong (β = 0.554, *p* < 0.001; 95% BC CI: [0.436, 0.648]). This pattern is consistent with the proposition that the social environment has an association with continuation intention that is not statistically accounted for by pressure self-efficacy alone. It should not, however, be interpreted as proof of a direct causal process. Unmeasured factors—such as competitive level, remuneration, abuse exposure, organisational identification and job satisfaction—may contribute to both perceived climate and continuation intention. Adjustment for the available covariates (age, sex, income and officiating experience) did not change the pattern (Table 9), but residual confounding by unmeasured variables cannot be excluded.

Respect climate was also positively associated with self-efficacy for performing under pressure (β = 0.276, *p* < 0.001; 95% BC CI: [0.192, 0.363]). Higher PRS scores indicate perceived capability to remain effective when facing pressure from players, spectators and coaches, rather than greater exposure to psychological pressure. The observed association is, therefore, compatible with the idea that supportive social contexts and efficacy beliefs co-occur [20,60,61], but the same-wave design cannot determine whether a more respectful climate strengthens efficacy, more efficacious officials perceive their environment differently, or both are influenced by third variables.

Pressure self-efficacy also showed a positive but comparatively small association with continuation intention (β = 0.117, *p* = 0.008; 95% BC CI: [0.029, 0.204]). This finding is consistent with evidence that referee efficacy is related to coping and functional performance [21,62,63], while underscoring that continuation intention is unlikely to be explained by individual psychological resources alone. In practical terms, efficacy-focused training may be useful, but the present data do not establish that strengthening pressure self-efficacy will cause referees to remain active.

The model-based indirect association through pressure self-efficacy was statistically detectable but small (β = 0.032; 95% BC CI: [0.010, 0.064]) and represented 5.5% of the total association. Under ordinal WLSMV estimation, the indirect association was larger (β = 0.071, 95% CI: [0.034, 0.108]; 11.4% of the total), so its magnitude is estimator-dependent, although under both estimators, it was small relative to the direct association. The poorer fit of models constraining either the direct RRC–RCI path or the PRS–RCI path to zero supports retaining both paths within the specified covariance structure. Because the structural part of the model is just-identified, its global fit is identical to that of the measurement model and cannot adjudicate between alternative orderings of the constructs [52]. It does not, however, verify a temporal mediating mechanism. A stronger test would require repeated measurement establishing that perceived respect climate precedes change in pressure self-efficacy and that such change predicts subsequent continuation behaviour, ideally with alternative longitudinal models compared directly.

The additional robustness analyses strengthen the statistical interpretation without changing its causal limits. The WLSMV re-estimation produced the same ordering of effects as the primary robust-ML model, indicating that the central pattern was not an artefact of treating the ordered indicators as continuous, and adjustment for age, sex, income and officiating experience, as well as alternative RRC item sets, left the pattern unchanged. Loading and intercept invariance supported comparison of the latent constructs across men and women, but ordinal threshold invariance was only approximate. Likelihood-ratio equality tests suggested that the PRS → RCI path and the indirect product were larger among women, whereas Wald-type confidence intervals for the between-group differences marginally included zero. This exploratory result raises the possibility that perceived competence under pressure may have greater relevance to continuation intention among women referees, but its post hoc nature and the smaller female subgroup mean that it should be considered a replication target rather than a definitive moderator effect.

Taken together, the results suggest that perceived respect climate is the more prominent correlate of continuation intention in the present model, whereas pressure self-efficacy contributes a smaller complementary association. This interpretation is compatible with research emphasising social support, belonging, organisational commitment and positive working climates in sport-officiating sustainability [64,65,66,67,68]. For organisations, the findings identify respect climate as a plausible target for future intervention research rather than demonstrating that any specific organisational practice will increase retention. Prospective studies should test whether changes in respect climate are followed by measurable changes in both continuation intentions and verified officiating participation.

The model explained 35.6% of the variance in continuation intention (R^2^ = 0.356) and showed good global fit (robust CFI = 0.991, robust TLI = 0.989, robust RMSEA = 0.032 [0.014, 0.047], SRMR = 0.034); because the structural part is just-identified, this fit reflects the measurement model. These values indicate that the specified covariance structure is compatible with the observed data, not that it is uniquely correct. Substantial unexplained variance remains, and key contextual variables were unavailable or unsuitable for covariate adjustment, including competitive level, remuneration and abuse exposure; officiating experience could be classified for only 579 participants because of the online-form problem, although adjusting for it did not alter the findings. Accordingly, the practical contribution of this study is best framed as identifying a potentially important social-context correlate and providing instruments for further testing, rather than as establishing a determinant of actual referee retention.

Several limitations define the scope of inference. First, the cross-sectional design precludes temporal or causal conclusions about the ordering of respect climate, pressure self-efficacy, and continuation intention; the indirect path should, therefore, be interpreted as a statistical decomposition of same-wave associations rather than as evidence of established mediation. Second, convenience sampling and online self-report may limit representativeness and generalisability across sports, competitive levels, and national officiating systems. Third, the RRC and RCI were developed and evaluated within the same overall study. Although calibration/validation splitting reduced some risk of overfitting, neither measure should be considered fully validated without replication in independent samples. Moreover, the removal of one RRC item was initially informed by the full sample. The repeated-split analysis showed that the same item was selected in most calibration halves and that the five-item form met the stated fit criteria in most held-out halves, but all splits came from the same overall sample; this is an internal stability check rather than external validation, and the RRC structure remains provisional. Additional limitations concern measurement and analysis. Approximately one-third of participants did not report a sport or were classified under other/individual-sport responses, and the officiating-experience item contained 45 placeholder-labelled responses and one blank response; these issues prevented sport-adjusted analyses and restricted experience-adjusted analyses to 579 participants. RRC AVE (0.484) was marginally below 0.50, indicating borderline convergent evidence. Content review of the newly developed measures relied on qualitative judgements of three experts, and quantitative content-validity indices (CVI/CVR) were not calculated; a three-member panel is at the lower bound for computing such indices, and the sources that guided the expert review [27,28] recommend reporting them, so this remains a limitation of the present development work. No separate referee pilot study or pretest was conducted for either new scale; therefore, pre-field evidence is limited to the qualitative expert review. In addition, active referee status was ensured through the distribution channel rather than verified against individual licence records. The positive-versus-negative split among the original RCI candidate items may partly reflect wording-related method variance. The RCI also includes items on personal meaning, career progression and willingness to take on more matches; although these loaded strongly on the same factor as the plan-to-continue items, the scale may overlap with occupational commitment, and its discriminant validity against commitment measures has yet to be established. Finally, the PRS items were administered in the four-category format of the final REFS. The three PRS items showed high item-level collinearity (outer VIF up to 5.63), and the size of the small indirect association differed between ML and ordinal estimation. The initial RRC pool also comprised only six items, which is smaller than the pools recommended in scale-development guidance [28]; the measure is, therefore, best read as a brief, context-specific index of perceived respect climate, developed for settings in which questionnaire space is limited, rather than as a fully developed scale, and the item pool needs to be expanded before its structure can be regarded as settled. The Turkish RRC3 item administered to participants is double-barrelled, combining the restraint of crowd reactions with their independence from the referee’s decisions. RRC3 carried the lowest standardised loading of all fourteen indicators in the measurement model (0.642) and the lowest communality in the exploratory solution (h^2^ = 0.404), and it should be split into two separate items in any further development of the measure.

## 5. Conclusions

This cross-sectional study found a strong positive association between perceived referee respect climate and intention to continue refereeing, together with a smaller statistical indirect association through self-efficacy for performing under pressure. The structural pattern was preserved in ordinal (WLSMV), covariate-adjusted and alternative-item sensitivity analyses, although the two point estimates differed (β = 0.032 under robust ML and β = 0.071 under WLSMV); their confidence intervals are reported separately in Section 3. Loading and intercept invariance supported comparison across sex, with approximate threshold invariance. Exploratory multigroup tests suggested a larger PRS → RCI path and indirect component among women, a test-dependent finding that requires independent replication. The RRC and RCI provide brief referee-specific measures with promising initial psychometric evidence, but independent validation and longitudinal follow-up are required before stronger conclusions about actual referee retention can be drawn.

Future studies should validate the RRC and RCI in independent and more diverse referee samples, including different sports, competitive levels, and national contexts. Longitudinal designs are needed to clarify the temporal relationships among respect climate, pressure self-efficacy, and continuation intention and to determine whether the observed indirect association reflects a genuine mediating process. Further research should also incorporate objective indicators of referee retention, use prospectively specified robust ordinal SEM approaches, compute quantitative content-validity indices for the new measures, test the discriminant validity of the RCI against occupational-commitment measures, and account for relevant individual and organisational factors such as officiating experience, referee level, match exposure, remuneration, and organisational support. The exploratory sex-related structural differences should be replicated in larger and more balanced samples.

## Figures and Tables

**Table 1:** Participant characteristics (*N* = 625).

Variable	Category	*n*	%
Sex	Women	202	32.3
	Men	423	67.7
Age (years)	M = 33.56, SD = 10.35, range: 18–65	625	100.0
Income (monthly, TRY)	0–23,000	161	25.8
	23,001–46,000	96	15.4
	46,001–69,000	172	27.5
	69,001 or more	196	31.4
Sport	Volleyball	184	29.4
	Basketball	97	15.5
	Football	87	13.9
	Handball	52	8.3
	Other sports (mainly individual)	17	2.7
	Not reported (blank)	188	30.1
Officiating experience	1 year	58	9.3
	2 years	46	7.4
	3 years	80	12.8
	4 years or more	395	63.2
	Unclassifiable: placeholder label “Option 1”	21	3.4
	Unclassifiable: placeholder label “Option 2”	24	3.8
	Blank	1	0.2
Province of residence	63 provinces represented (624 reporting; 1 blank)	—	—

Note. The raw export recorded the officiating-experience categories “1”, “2”, “3” and “4 ve üzeri” (“4 or more”) years, together with two options that retained the survey platform’s default placeholder labels (“1. seçenek”/“Option 1” (*n* = 21); “2. seçenek”/“Option 2” (*n* = 24)) and one blank response. Because the intended meaning of the two placeholder labels cannot be recovered, the 45 placeholder-labelled responses and the one blank response were treated as unavailable for experience-adjusted analyses; 579 participants had classifiable experience data. An em dash (—) indicates that a separate frequency or percentage is not applicable to the summary of provinces represented. The response labels reported here follow the predefined categories in the raw export. All other questionnaire items were checked against their predefined response labels in the raw export, and no comparable problem was found.

**Table 2:** Item statistics and EFA/CFA factor loadings for the RRC and RCI scales.

Scale/Item	M	SD	Skew.	Kurt.	r (Item–Total)	EFA Loading	h^2^	CFA Loading	α If Deleted	VIF
*Referee Respect Climate (RRC)*										
RRC1 (orig. item 1)	4.10	0.94	−1.34	1.84	0.65	0.802	0.643	0.735	0.773	1.76
RRC2 (orig. item 3)	4.11	0.89	−1.37	2.33	0.65	0.688	0.474	0.738	0.773	1.79
RRC3 (orig. item 4)	3.92	1.09	−1.03	0.38	0.58	0.636	0.404	0.642	0.797	1.53
RRC4 (orig. item 5)	3.77	0.97	−0.79	0.27	0.64	0.715	0.511	0.711	0.777	1.69
RRC5 (orig. item 6)	4.18	0.83	−1.47	3.32	0.56	0.638	0.408	0.647	0.800	1.49
*Referee Continuation Intention (RCI)*										
RCI1	4.32	0.94	−1.62	2.58	0.79	0.830	0.689	0.840	0.907	2.87
RCI2	4.40	0.80	−1.68	3.64	0.81	0.855	0.731	0.849	0.905	3.04
RCI3	4.45	0.83	−1.99	4.78	0.69	0.706	0.499	0.732	0.920	2.07
RCI4	4.22	0.95	−1.42	2.02	0.76	0.781	0.610	0.801	0.912	2.49
RCI5	4.40	0.88	−1.85	3.84	0.83	0.847	0.718	0.865	0.902	3.35
RCI6	4.42	0.86	−1.91	4.13	0.79	0.825	0.680	0.827	0.907	2.77
*Pressure Subscale (PRS)*										
PRS1	3.24	0.96	−1.18	0.40	0.86	—	—	0.889	0.937	3.85
PRS2	3.29	0.96	−1.30	0.67	0.91	—	—	0.952	0.901	5.63
PRS3	3.26	0.96	−1.19	0.35	0.89	—	—	0.925	0.916	4.90

Note. Means, SDs, skewness, kurtosis, item–total correlations and alpha values were computed in the total analytic sample (*n* = 625). EFA loadings and communalities (h^2^) were obtained by principal axis factoring in the seed-documented calibration half (*n* = 312; seed 20260921). CFA loadings are standardised estimates from the three-factor measurement model (*n* = 625); all loadings are significant at *p* < 0.001. The pressure subscale was drawn from a previously validated instrument and was not entered into the EFA. Original RRC item 2 was removed because of local dependence. VIF = item-level (outer) variance inflation factor, obtained by regressing each item on the other items of the same construct in the total sample; values < 5 are commonly regarded as unproblematic [38]. The value of 5.63 for PRS2 reflects the near-parallel content of the three pressure items (pressure from players, spectators and coaches) within one construct; the construct-level (inner) VIF was 1.06.

**Table 3:** Reliability, convergent and discriminant validity (*N* = 625).

Construct	Items	Loading Range	α	CR	AVE	√AVE	1	2	3
1. Referee respect climate (RRC)	5	0.642–0.738	0.820	0.824	0.484	0.696	(0.696)	0.601 [0.509, 0.683]	0.279 [0.194, 0.363]
2. Continuation intention (RCI)	6	0.732–0.865	0.923	0.925	0.672	0.820	0.586	(0.820)	0.281 [0.194, 0.365]
3. Pressure self-efficacy (PRS)	3	0.889–0.952	0.944	0.945	0.850	0.922	0.277	0.270	(0.922)

Note. CR = composite reliability; AVE = average variance extracted. Values below the diagonal are latent correlations from the measurement model; values above the diagonal are HTMT ratios with 95% percentile-bootstrap confidence intervals in brackets (5000 resamples). Diagonal values in parentheses are the square roots of AVE. All correlations are significant at *p* < 0.001.

**Table 4:** Descriptive statistics, normality, correlations and multicollinearity diagnostics (*N* = 625).

Variable	M	SD	Skew.	Kurt.	1	2	3	VIF	Tolerance
1. Referee respect climate	4.02	0.72	−1.12	2.14	—			1.06	0.94
2. Continuation intention	4.37	0.75	−1.74	4.13	0.52 ***	—		—	—
3. Pressure self-efficacy	3.26	0.91	−1.28	0.72	0.24 ***	0.26 ***	—	1.06	0.94

Note. Respect climate and continuation intention are scored 1–5; pressure self-efficacy is scored 1–4. VIF and tolerance were computed for the predictors only (respect climate and pressure self-efficacy), with continuation intention as the dependent variable. *** *p* < 0.001.

**Table 5:** Direct, indirect and total model-based associations: unstandardised robust-ML and standardised bootstrap estimates (N = 625).

Path	B	SE	z	*p*	β	Boot SE	95% BC CI (β)	Hypothesis
*Direct effects*								
RRC → RCI (c′)	0.635	0.081	7.81	<0.001	0.554	0.053	[0.436, 0.648]	H1 supported
RRC → PRS (a)	0.340	0.061	5.60	<0.001	0.276	0.044	[0.192, 0.363]	H2 supported
PRS → RCI (b)	0.109	0.041	2.66	0.008	0.117	0.044	[0.029, 0.204]	H3 supported
*Indirect effect*								
RRC → PRS → RCI (a × b)	0.037	0.015	2.42	0.016	0.032	0.014	[0.010, 0.064]	H4 supported
*Total effect*								
RRC → RCI (c′ + a × b)	0.673	0.077	8.73	<0.001	0.586	0.047	[0.486, 0.671]	—

Note. B = unstandardised estimate; SE = robust (Satorra–Bentler) standard error; z = B/SE, computed from the unrounded estimates, so recomputing z from the three-decimal values printed here can differ in the second decimal; the unstandardised estimates are likewise rounded independently, so the printed total effect can differ by 0.001 from the sum of the printed direct and indirect estimates; *p* = two-sided *p*-value based on the robust SE (for a × b and the total effect, delta-method robust SE); β = standardised coefficient; Boot SE = bootstrap standard error of β; BC CI = bias-corrected bootstrap confidence interval for β (5000 resamples; seed 20260921). Statistical evidence for indirect and total associations was evaluated by whether the confidence interval excluded zero. The indirect/total ratio was 5.5%. R^2^(RCI) = 0.356; R^2^(PRS) = 0.076. Because all constructs were measured cross-sectionally, the indirect estimate should not be interpreted as evidence of temporal or causal mediation.

**Table 6:** Comparison of competing structural models (*N* = 625).

Model	ML χ^2^	df	Scaled χ^2^	CFI (Robust)	TLI (Robust)	RMSEA (Robust)	SRMR	Scaled Δχ^2^	Δdf	*p*
M1. Hypothesised model (a, b and c′ free)	178.61	74	101.66	0.991	0.989	0.032	0.034	—	—	—
M2. Direct path constrained (c′ = 0)	340.48	75	192.70	0.963	0.955	0.067	0.161	64.58	1	<0.001
M3. PRS → RCI path constrained (b = 0)	187.86	75	107.23	0.990	0.988	0.035	0.047	6.66	1	0.010

Note. Estimation by robust ML (MLM). Scaled Δχ^2^ = Satorra–Bentler (2001) scaled chi-square difference test against M1 [45]; the corresponding normal-theory ML differences were Δχ^2^(1) = 161.87 (M2) and 9.25 (M3). Both constrained alternatives fit significantly worse than M1. Because M1 is just-identified in its structural part, its fit equals that of the measurement model.

**Table 7:** Multigroup measurement invariance tests by sex under robust ML (**A**) and ordinal WLSMV estimation (**B**) (N = 625).

Model	Scaled χ^2^ (df)	CFI	TLI	RMSEA	SRMR	Scaled Δχ^2^ (Δdf)	*p*	ΔCFI
(**A**) Robust ML (responses treated as continuous)								
Configural	177.23 (148)	0.991	0.989	0.032	0.039	—	—	—
Metric (loadings)	182.51 (159)	0.993	0.992	0.028	0.042	5.58 (11)	0.900	+0.002
Scalar (loadings + intercepts)	196.71 (170)	0.992	0.992	0.028	0.043	15.40 (11)	0.165	−0.001
(**B**) WLSMV (responses treated as ordinal)								
Configural	262.77 (148)	0.995	0.994	0.050	0.042	—	—	—
Thresholds	287.76 (162)	0.994	0.994	0.050	0.042	29.58 (14)	0.009	−0.001
Thresholds + loadings	286.94 (173)	0.995	0.995	0.046	0.042	5.88 (11)	0.882	+0.001
Thresholds + loadings + intercepts	295.53 (184)	0.995	0.995	0.044	0.042	12.43 (11)	0.332	0.000

Note. Each model was compared with the immediately preceding model. (A) Maximum likelihood with Satorra–Bentler scaled statistics; CFI, TLI and RMSEA are robust versions; normal-theory ML χ^2^ = 289.39, 298.88 and 312.77 for the configural, metric and scalar models, and the configural value equals the sum of the separately estimated group values (158.255 + 131.137 = 289.392). (B) WLSMV with theta parameterisation and the identification conditions of Wu and Estabrook [49]; response categories 1 and 2 of the five-point items were collapsed because some categories were empty in the female group (e.g., RCI2); CFI, TLI and RMSEA are scaled versions; Δχ^2^ values are scaled–shifted difference tests.

**Table 8:** Sex-specific structural estimates from the scalar-invariant multigroup model (reference model; men n = 423, women n = 202).

Path	Men: B [95% CI]	*p*	Men: β [95% CI]	Women: B [95% CI]	*p*	Women: β [95% CI]	Equality Test: Scaled Δχ^2^(1), *p*
RRC → PRS (a)	0.325 [0.200, 0.451]	<0.001	0.284 [0.187, 0.381]	0.402 [0.103, 0.700]	0.008	0.275 [0.097, 0.454]	0.27, 0.605
RRC → RCI (c′)	0.638 [0.455, 0.822]	<0.001	0.579 [0.460, 0.698]	0.632 [0.371, 0.893]	<0.001	0.490 [0.286, 0.694]	<0.01, 0.967
PRS → RCI (b)	0.060 [−0.042, 0.163]	0.245	0.063 [−0.043, 0.169]	0.219 [0.095, 0.342]	0.001	0.247 [0.107, 0.388]	6.86, 0.009
RRC → PRS → RCI (a × b)	0.020 [−0.014, 0.054]	0.254	0.018 [−0.013, 0.048]	0.088 [0.001, 0.174]	0.046	0.068 [0.005, 0.131]	8.55, 0.003

Note. Estimates from the robust-ML multigroup model with equal loadings and intercepts (scalar-invariant model), which served as the reference model for all between-group comparisons. B = unstandardised estimate; β = standardised estimate; 95% CIs and *p*-values are based on robust (Satorra–Bentler) standard errors (delta method for a × b). Equality tests constrain the unstandardised parameter (or the product a × b) to be equal across groups and compare the result with the reference model. Wald-type 95% CIs for the between-group differences, computed from the unrounded estimates of this model, were ΔB = 0.158 [−0.001, 0.317] for PRS → RCI and 0.068 [−0.025, 0.161] for a × b. R^2^(RCI) = 0.360 (men) and 0.368 (women); R^2^(PRS) = 0.081 and 0.076.

**Table 9:** Standardised structural estimates across sensitivity analyses.

Specification	n	RRC → PRS (a)	PRS → RCI (b)	RRC → RCI (c′)	a × b	R^2^ (RCI)	CFI/RMSEA
1. Primary model: robust ML, five-item RRC	625	0.276 [0.191, 0.361]	0.117 [0.031, 0.203]	0.554 [0.451, 0.657]	0.032 [0.006, 0.058]	0.356	0.991/0.032
2. Ordinal WLSMV	625	0.404 [0.324, 0.483]	0.176 [0.089, 0.262]	0.554 [0.478, 0.630]	0.071 [0.034, 0.108]	0.416	0.995/0.048
3. Adjusted for age, sex and income	625	0.269 [0.183, 0.354]	0.117 [0.032, 0.203]	0.556 [0.453, 0.659]	0.032 [0.006, 0.057]	0.362	0.986/0.035
4. Model 3 + officiating experience	579	0.246 [0.155, 0.337]	0.105 [0.016, 0.194]	0.570 [0.462, 0.679]	0.026 [0.002, 0.050]	0.368	0.985/0.034
5. Unadjusted, same subsample as model 4	579	0.267 [0.178, 0.357]	0.102 [0.012, 0.192]	0.562 [0.453, 0.670]	0.027 [0.001, 0.053]	0.356	0.994/0.028
6. Six-item RRC (all candidate items)	625	0.262 [0.177, 0.346]	0.132 [0.047, 0.217]	0.525 [0.427, 0.623]	0.035 [0.009, 0.060]	0.330	0.978/0.050
7. Five-item RRC without original item 6	625	0.255 [0.169, 0.340]	0.143 [0.057, 0.229]	0.498 [0.401, 0.595]	0.036 [0.011, 0.062]	0.305	0.986/0.043

Note. Values are standardised coefficients with 95% delta-method confidence intervals based on robust (models 1 and 3–7) or WLSMV (model 2) standard errors; bias-corrected bootstrap intervals for the primary model are reported in Table 5. CFI and RMSEA are robust (models 1, 3–7) or scaled (model 2) values. In models 3 and 4, covariates predicted RRC, PRS and RCI; age was entered in decades, sex as women = 1, and income as four ordered bands and experience as four ordered categories (1, 2, 3 and ≥4 years). Full covariate estimates are given in Table S4. Model 4 coded classifiable officiating experience ordinally as 1, 2, 3 and 4 for 1, 2, 3 and ≥4 years, respectively.

## Data Availability

The data presented in this study are available from the corresponding author upon reasonable request. The data are not publicly available due to privacy and confidentiality considerations relating to the protection of participants’ individual-level information. Data S1 contains the R analysis script and model syntax (Data_S1_analysis_script.R) and the case-level calibration/validation membership file (Data_S1_split_assignment.csv); the documented split/resampling seed is 20260921. Reporting was prepared with reference to the STROBE recommendations for cross-sectional studies. Psychometric findings for the newly developed RRC and RCI are explicitly presented as initial evidence rather than definitive validation. Because the questionnaire link was distributed through referee-board WhatsApp groups rather than to an enumerated list, the number of referees who received it could not be determined, and a response rate cannot be computed; the 630 responses, therefore, constitute a convenience sample of unknown coverage.

## Supplementary Materials

The following supporting information can be downloaded at https://www.mdpi.com/article/10.3390/healthcare14203476/s1. Table S1: Full oblimin-rotated EFA of the ten candidate RCI items (calibration half); Table S2: Repeated random-split evaluation of RRC item reduction and of the RCI (1000 splits); Table S3: Validation-half CFAs of the six- and five-item RRC and the six-item RCI; Table S4: Full covariate-adjusted structural models; Data S1: Data_S1_analysis_script.R (R script and model syntax) and Data_S1_split_assignment.csv (case-level calibration/validation membership; seed 20260921).

## Abbreviations

AVE, average variance extracted; BC CI, bias-corrected confidence interval; CFA, confirmatory factor analysis; CFI, comparative fit index; CR, composite reliability; DWLS, diagonally weighted least squares; EFA, exploratory factor analysis; HTMT, heterotrait–monotrait ratio; ML, maximum likelihood; MLM, maximum likelihood with Satorra–Bentler scaled statistics and robust standard errors; PRS, pressure self-efficacy; RCI, Referee Continuation Intention Scale; REFS, Referee Self-Efficacy Scale; RRC, Referee Respect Climate Scale; RMSEA, root-mean-square error of approximation; SEM, structural equation modelling; SRMR, standardized root-mean-square residual; TLI, Tucker–Lewis index; VIF, variance inflation factor; WLSMV, weighted least squares, mean- and variance-adjusted.

## Appendix A. Items of the Developed Scales

**Table A1:** Referee Respect Climate Scale (RRC)—Turkish Original and English Translation.

Code	Turkish (Original)	English Translation	Status
RRC1	Maçlarda genel olarak bana saygılı bir iletişim kurulur.	In matches, people generally communicate with me respectfully.	Retained
—	Oyuncular itiraz etse bile sınırı aşmaz.	Even when players protest, they do not overstep the line.	Removed (local dependence)
RRC2	Antrenörlerle konuşmalarım genelde sakin ve yapıcı geçer.	My conversations with coaches are generally calm and constructive.	Retained
RRC3	Tribün tepkileri kararlarımdan bağımsız olarak ölçülüdür.	Reactions from the stands remain restrained, regardless of the decisions I make.	Retained
RRC4	Saha içi gerilim çıktığında hızla yatışır.	When tension arises on the field of play, it subsides quickly.	Retained
RRC5	Maç görevlileri (gözlemci/dördüncü hakem vb.) beni destekleyici bir tavırdadır.	Match officials (observers, fourth officials, etc.) are supportive towards me.	Retained

Note. Responses are given on a five-point Likert scale (1 = strongly disagree; 5 = strongly agree). No items are reverse-scored. The English translation of RRC3 reflects the intended meaning of the Turkish item, which states that spectators’ reactions stay within reasonable limits irrespective of the referee’s decisions.

**Table A2:** Referee Continuation Intention Scale (RCI) and Pressure Subscale (PRS) Items.

Code	Turkish (Original)	English Translation
RCI1	Hakemliği uzun yıllar sürdürmek istiyorum.	I want to continue refereeing for many years.
RCI2	Hakemlik benim için anlamlı bir parça.	Refereeing is a meaningful part of my life.
RCI3	Zor maçlar bile bırakma isteğimi artırmaz.	Even difficult matches do not increase my desire to quit.
RCI4	Kendimi hakemlikte ilerlerken görüyorum.	I see myself progressing in refereeing.
RCI5	Yeni sezonda da aktif olmak isterim.	I would like to be active again next season.
RCI6	Vakit buldukça daha çok maç almak isterim.	I would like to take on more matches whenever I have time.
PRS1	Oyuncular tarafından yapılan baskıdan etkilenmeme	Not being affected by pressure from players
PRS2	Seyirci tarafından yapılan baskıdan etkilenmeme	Not being affected by pressure from spectators
PRS3	Antrenörler tarafından yapılan baskıdan etkilenmeme	Not being affected by pressure from coaches

Note. RCI items are rated 1–5 (strongly disagree to strongly agree). PRS items are drawn from the pressure subscale of the Referee Self-Efficacy Scale [21] in the 13-item Turkish form examined by Unutmaz and Gençer [30] and were administered in the raw survey on a four-category scale coded 1 = low, 2 = moderate, 3 = high, and 4 = complete. The four attrition-keyed candidate items included in the survey form are listed in Table A3.

**Table A3:** Candidate attrition-keyed items administered but excluded from the RCI.

Code	Turkish (Original)	English Translation	EFA Loading (Factor 2)
ATT1	Maçlardan sonra zihnen tükenmiş hissederim.	I feel mentally exhausted after matches.	0.488
ATT2	Son dönemde hakemlik beni yıpratıyor.	Lately, refereeing has been wearing me down.	0.894
ATT3	Hakemlikten soğuduğumu düşünüyorum.	I think I have lost my enthusiasm for refereeing.	0.893
ATT4	İlk fırsatta hakemliği bırakmayı planlıyorum.	I plan to quit refereeing at the first opportunity.	0.587

Note. Rated 1–5 (strongly disagree to strongly agree). Loadings are oblimin-rotated principal-axis pattern coefficients on the attrition factor in the calibration half (Table S1). These items were not reverse-scored into the RCI and were not used in the structural model.
