# Supplementary material for: Perceived Respect Climate, Pressure Self-Efficacy, and Intention to Continue Refereeing: Scale Development and a Cross-Sectional Structural Equation Model
Source: Healthcare (Basel). 2026 Oct 16;14(20):3476. doi: 10.3390/healthcare14203476 (PMC13704062; doi:10.3390/healthcare14203476)
Supplement: Supplementary file 1 [file healthcare-14-03476-s001.zip › healthcare-4572129-supplementary.pdf]

Supplementary Materials

Perceived Respect Climate, Pressure Self-Efficacy, and Intention to Continue Refereeing: Scale Development and a Cross-Sectional Structural Equation Model

Manuscript ID: healthcare-4572129

Contents. Table S1 — full exploratory factor analysis of the ten candidate continuation/attrition items in the calibration half. Table S2 — repeated random-split evaluation of the Referee Respect Climate (RRC) item-reduction decision and of the six-item Referee Continuation Intention (RCI) scale (1000 splits). Table S3 — validation-half confirmatory factor analyses of the six- and five-item RRC and of the six-item RCI. Table S4 — complete covariate-adjusted structural models. Data S1 — analysis script (Data_S1_analysis_script.R) and calibration/validation membership (Data_S1_split_assignment.csv).

All covariance-structure analyses were estimated by maximum likelihood with Satorra–Bentler scaled test statistics and robust standard errors (MLM), except where ordinal (WLSMV) estimation is indicated. Robust CFI, TLI and RMSEA are reported throughout. The calibration/validation split and every resampling procedure used the seed 20260921.

Table S1. Exploratory factor analysis of the ten candidate continuation and attrition items, calibration half (n = 312).

Item	Factor 1 (continuation)	Factor 2 (attrition)	h^2
RCI1 I want to continue refereeing for many years.	.761	−.124	.702
RCI2 Refereeing is a meaningful part of my life.	.844	−.024	.736
RCI3 Even difficult matches do not increase my desire to quit.	.771	.109	.511
RCI4 I see myself progressing in refereeing.	.758	−.028	.600
RCI5 I would like to be active again next season.	.863	.026	.720
RCI6 I would like to take on more matches whenever I have time.	.826	.000	.682
ATT1 I feel mentally exhausted after matches.	−.102	.488	.305
ATT2 Lately, refereeing has been wearing me down.	.026	.894	.776
ATT3 I think I have lost my enthusiasm for refereeing.	.022	.893	.773
ATT4 I plan to quit refereeing at the first opportunity.	−.215	.587	.533

Note. Principal-axis factoring with oblimin rotation in the calibration half (n = 312; seed 20260921). Values are pattern coefficients. Parallel analysis against 1000 random data sets retained two factors. The factor correlation was r = −.57. The maximum absolute cross-loading was .215 (ATT4 = −.215). Sampling adequacy was satisfactory (KMO = .876; Bartlett's $\chi^2(45) = 2063.3$, $p < .001$). Sensitivity checks: a promax rotation reproduced the same two-factor pattern (continuation items .77–.87, attrition items .50–.91, largest cross-loading |.20|, factor correlation r = −.60), and a forced one-factor solution placed the six continuation items at .65 to .83 and the four attrition items at −.48 to −.66, accounting for 51.0% of the total variance. The six continuation-keyed items were retained as the operational RCI because they represent the intended construct; the attrition factor combines exhaustion and strain content with quitting plans and is conceptually distinct.

Table S2. Repeated random-split evaluation of the RRC item-reduction decision and of the six-item RCI (1000 repeated random splits within the same sample).

Panel A. Item identified for removal within the calibration half.

Original RRC item	% of 1000 calibration halves
Original item 1	0.9

Original item 2 — the item removed in the primary analysis	90.9
Original item 3	1.3
Original item 4	0.0
Original item 5	1.6
Original item 6	5.3

Panel B. Fit of the resulting scales in the held-out half within each repeated split.

Criterion	Five-item RRC	Six-item RCI
Splits meeting robust CFI ≥ .95 and SRMR ≤ .08 (%)	94.3	99.6
Median robust CFI	0.990	0.989
Median robust TLI	0.981	0.981
Median robust RMSEA	0.055	0.073
Median SRMR	0.026	0.027
5th percentile of robust CFI	0.947	0.966

Note. In each of 1000 repeated random splits of the same 625 analysed cases into halves of 312 and 313, the item-removal rule was re-applied within the calibration half: among the six possible single-item deletions, the deletion producing the lowest normal-theory ML χ^2 was selected (all candidate five-item models have df = 5). The resulting five-item form was then estimated in the corresponding held-out half, and the six-item RCI was evaluated in the same held-out half. All models are one-factor CFAs estimated by robust ML. The analysis is implemented in Data S1, Section 13. Original item 2, which was removed in the primary analysis, was identified in 90.9% of calibration halves, and the resulting five-item form met robust CFI ≥ .95 and SRMR ≤ .08 in 94.3% of held-out halves. These repeated splits are an internal stability analysis within the same participant sample; they do not constitute validation in a new sample and do not undo the original use of the full sample for item selection. In the single seed-documented split reported in the main text, the rule pointed to original item 6 rather than item 2 (reflecting a residual dependence between original items 3 and 6); structural models using the six-item pool or the alternative five-item form nevertheless reproduced the same substantive pattern (Table 9).

Table S3. Confirmatory factor analyses of the RRC and RCI in the seed-documented validation half.

Model	Sample	ML χ^2 (df)	Scaled χ^2	CFI_r	TLI_r	$RMSEA_r$ [90% CI]	SRMR
Six-item RRC (all candidate items)	Full sample (625)	88.46 (9)	54.25	.950	.917	.115 [.086, .145]	.041
Six-item RRC (all candidate items)	Validation (313)	71.73 (9)	42.23	.923	.872	.142 [.100, .186]	.053
Five-item RRC (final form)	Validation (313)	15.25 (5)	10.92	.983	.967	.073 [.000, .132]	.028
Six-item RCI	Validation (313)	39.25 (9)	14.09	.990	.983	.071 [.000, .139]	.024

Note. One-factor models estimated by robust ML (MLM). Subscript r denotes robust (Satorra–Bentler-corrected) fit indices. The six-item RRC was first estimated in the full analytic sample, where poor fit and local dependence prompted removal of original item 2 (the single-item deletion with the lowest ML χ^2 and highest robust CFI); the two held-out-half rows from the seed-documented split show that the six-item form did not fit acceptably whereas the five-item form did; this within-sample split does not constitute external validation. Split membership is given in Data_S1_split_assignment.csv.

Table S4. Complete covariate-adjusted structural models.

Model A. Adjusted for age, sex and income (n = 625).

Parameter	B	Robust SE	95% CI (B)	β	p
**Structural paths**					
RRC → PRS (a)	0.330	0.060	[0.213, 0.448]	.269	< .001
RRC → RCI (c′)	0.637	0.081	[0.478, 0.795]	.556	< .001
PRS → RCI (b)	0.109	0.041	[0.029, 0.189]	.117	.007
**Covariate associations**					
Age (decades) → RRC	−0.092	0.033	[−0.156, −0.028]	−.138	.005
Age (decades) → PRS	−0.095	0.048	[−0.189, −0.002]	−.116	.046
Age (decades) → RCI	−0.015	0.034	[−0.082, 0.051]	−.020	.654
Women (vs. men) → RRC	0.084	0.065	[−0.044, 0.211]	.057	.199
Women (vs. men) → PRS	−0.178	0.075	[−0.325, −0.030]	−.098	.018
Women (vs. men) → RCI	−0.023	0.059	[−0.138, 0.092]	−.014	.694
Income band → RRC	0.084	0.033	[0.020, 0.148]	.142	.010
Income band → PRS	0.056	0.038	[−0.020, 0.131]	.077	.147
Income band → RCI	−0.047	0.032	[−0.110, 0.016]	−.069	.144
Indirect association a × b	0.036	0.015	[0.007, 0.065]	.032	.016

Robust CFI = .986, robust TLI = .982, robust RMSEA = .035, SRMR = .032; R^2 = .362 (RCI), .095 (PRS), .016 (RRC).

Model B. Additionally adjusted for officiating experience (n = 579 with classifiable experience).

Parameter	B	Robust SE	95% CI (B)	β	p
**Structural paths**					
RRC → PRS (a)	0.301	0.062	[0.179, 0.423]	.246	< .001
RRC → RCI (c′)	0.652	0.086	[0.482, 0.821]	.570	< .001
PRS → RCI (b)	0.098	0.043	[0.015, 0.182]	.105	.021
**Covariate associations**					
Age (decades) → RRC	−0.137	0.034	[−0.204, −0.070]	−.203	< .001
Age (decades) → PRS	−0.136	0.050	[−0.235, −0.038]	−.166	.006
Age (decades) → RCI	0.006	0.037	[−0.066, 0.078]	.008	.865
Women (vs. men) → RRC	0.088	0.069	[−0.047, 0.223]	.058	.200
Women (vs. men) → PRS	−0.170	0.080	[−0.326, −0.014]	−.091	.033
Women (vs. men) → RCI	−0.052	0.061	[−0.172, 0.069]	−.030	.401
Income band → RRC	0.044	0.035	[−0.025, 0.113]	.072	.212
Income band → PRS	0.021	0.042	[−0.061, 0.103]	.028	.614
Income band → RCI	−0.034	0.035	[−0.102, 0.034]	−.049	.329
Officiating experience → RRC	0.109	0.037	[0.036, 0.182]	.156	.004
Officiating experience → PRS	0.088	0.045	[0.000, 0.177]	.104	.050
Officiating experience → RCI	−0.070	0.037	[−0.141, 0.002]	−.087	.058
Indirect association a × b	0.030	0.014	[0.002, 0.057]	.026	.036

Robust CFI = .985, robust TLI = .982, robust RMSEA = .034, SRMR = .032; R^2 = .368 (RCI), .100 (PRS), .035 (RRC).

Note. Estimation by robust ML (MLM); B = unstandardised estimate, β = standardised estimate, CIs and p values based on robust (Satorra–Bentler) standard errors (delta method for a × b). Age was entered in decades, sex as women = 1, income as four ordered bands, and officiating experience as four ordered categories (1, 2, 3 and 4 or more years). Covariates were allowed to predict all three latent constructs. In Model B the 45 placeholder-labelled and one blank experience responses were excluded. Standardised structural estimates for these models also appear as models 3 and 4 of Table 9 in the main text.
